# Genomic Investigation of Virulence Potential in Shiga Toxin *Escherichia coli* (STEC) Strains From a Semi-Hard Raw Milk Cheese

**DOI:** 10.3389/fmicb.2020.629189

**Published:** 2021-02-01

**Authors:** Claudia Cortimiglia, Maria Francesca Borney, Daniela Bassi, Pier Sandro Cocconcelli

**Affiliations:** Dipartimento di Scienze e Tecnologie Alimentari per una Filiera Agro-Alimentare Sostenibile (DISTAS), Università Cattolica del Sacro Cuore, Piacenza, Italy

**Keywords:** whole genome sequencing, STEC, virulence, LEE negative, raw milk cheese

## Abstract

Shiga-toxin-producing *Escherichia coli* (STEC) represents a significant cause of foodborne disease. In the last years, an increasing number of STEC infections associated with the consumption of raw and pasteurized milk cheese have been reported, contributing to raise the public awareness. The aim of this study is to evaluate the main genomic features of STEC strains isolated from a semi-hard raw milk cheese, focusing on their pathogenic potential. The analysis of 75 cheese samples collected during the period between April 2019 and January 2020 led to the isolation of seven strains from four *stx*-positive enrichment. The genome investigation evidenced the persistence of two serotypes, O174:H2 and O116:H48. All strains carried at least one *stx* gene and were negative for *eae* gene. The virulence gene pattern was homogeneous among the serogroup/ST and included adherence factors (*lpfA*, *iha*, *ompT*, *papC*, *saa*, *sab*, *hra*, and *hes*), enterohemolysin (*ehxA*), serum resistance (*iss*, *tra*), cytotoxin-encoding genes like *epeA* and *espP*, and the Locus of Adhesion and Autoaggregation Pathogenicity Islands (LAA PAIs) typically found in Locus of Enterocyte Effacement (LEE)-negative STEC. Genome plasticity indicators, namely, prophagic sequences carrying *stx* genes and plasmid replicons, were detected, leading to the possibility to share virulence determinants with other strains. Overall, our work adds new knowledge on STEC monitoring in raw milk dairy products, underlining the fundamental role of whole genome sequencing (WGS) for typing these unknown isolates. Since, up to now, some details about STEC pathogenesis mechanism is lacking, the continuous monitoring in order to protect human health and increase knowledge about STEC genetic features becomes essential.

## Introduction

Shiga toxin *Escherichia coli* (STEC) infections in the EU have increased in the last years, being the third most commonly reported zoonosis (EFSA and ECDC, 2019) and causing different types of diseases, ranging from uncomplicated diarrhea to hemorrhagic colitis and hemolytic–uremic syndrome (HUS) (Karmali, 2017). The STEC genome is characterized by high plasticity, making this group of organisms largely heterogeneous and difficult to classify in terms of pathogenicity (Bai et al., 2019). Currently, the serogroup is one of the factors used to identify STEC strains that have the capacity to cause human illness (Preueß et al., 2013). O157:H7 is the most frequently reported cause of severe STEC disease and outbreaks worldwide, but also the “top 6” non-O157 STEC serogroups (e.g., O26, O45, O103, O111, O121, and O145) have been identified as responsible for severe diseases and outbreaks. Moreover, other 250 STEC serotypes have been associated with human disease (Johnson and Nolan, 2009). Nevertheless, there are actually no criteria to accurately define STEC pathogenicity, and further knowledge of their virulence determinants is needed to improve the microbiological risk assessment. The STEC-associated risk lies in the presence of the main virulence factors Shiga toxins (Stx), classified in two forms, Stx1 and Stx2, each type being further categorized into four variants for Stx1 (a, c, d, and e) and 12 variants for Stx2 (a–i) (EFSA and ECDC, 2020). Among these subtypes, Stx2a is considered potentially related to a more severe illness. The *stx* genes are carried by lambdoid phages, which are highly mobile genetic elements, and, thanks to their mobility, the horizontal transfer and the dissemination, as much as the loss of the *stx* genes, are facilitated (Werber and Scheutz, 2019).

Although the pathogenic potential of STEC strains is strictly related to the presence of Shiga toxins, and therefore their detection is used as one of the first criteria for their classification, the capability to cause pathogenesis is also linked to other factors. In particular, it is well known that the risk for a severe disease increases if *stx2* gene is associated with *eae* gene on the Locus of Enterocyte Effacement (LEE). This gene codes for the intimin protein, which mediates the adhesion to the epithelial cells and the delivery of toxins, inducing the typical “attaching and effacing” lesion (Franzin and Sircili, 2015). Surprisingly, even if some of the major pathogenic strains are LEE-positive, it is noteworthy that also some LEE-negative strains were found to be associated with human disease (Galli et al., 2010; Krause et al., 2018; Colello et al., 2019). Indeed, they can produce other virulence factors, which are involved in alternative adherence mechanisms and which are encoded by plasmids or different Pathogenicity Islands (PAIs). In particular, STEC strains that do not host the LEE region were found to carry an alternative PAI called Locus of Adhesion and Autoaggregation (LAA), a four-module 86-kb mosaic region, which harbors adherence and autoaggregation factors potentially involved in pathogenicity mechanisms (Montero et al., 2017).

Whole genome sequencing (WGS) represents, nowadays, a powerful, affordable, and, by now, cheap technique for pathogenic bacteria identification and characterization, supporting the surveillance and the microbiological risk assessment and increasing the number of high-quality information to improve hazard identification (Collineau et al., 2019). Indeed, the availability of the almost entire genome leads to the analysis of potentially new pathogenic features and permits the evaluation of virulence profiles. In the latest years, many studies on STEC genomes were performed in order to investigate different aspects of their pathogenicity mechanisms and to predict the human health risk, considering both food and clinical isolates, in the attempt to define a useful scheme of classification (Ferdous et al., 2016; Lindsey et al., 2016; Njage et al., 2019; Sharma et al., 2019). The last European Food Safety Authority (EFSA) report about the pathogenicity assessment of STEC in food concludes that, up to now, a minimum genetic make-up required to cause human illness cannot be defined, but there are some factors related to an incremented probability to develop a disease (EFSA and ECDC, 2020).

A natural asymptomatic reservoir of STEC is recognized in cattle, representing a vehicle for human infections through direct contact or food products (EFSA and ECDC, 2020). Consequently, dairy products and, in particular, cheeses are documented to be associated with STEC infections (Farrokh et al., 2013; EFSA and ECDC, 2020). Currie et al. (2018) acknowledged an *Escherichia coli* O157:H7 outbreak linked to ripened Gouda cheese, observing a persistence of this strain during the production and the 60-day aging period (Currie et al., 2018). As well as Gouda cheese in Canada (Gill and Oudit, 2015), unpasteurized milk and cheese were also found to be a significative source of STEC contamination also in the United States (Costard et al., 2017) and recently in France (Bruyand et al., 2019). EFSA reported a total of 14 outbreaks linked to raw milk and raw milk products, confirming a tendency already observed in previous reports (EFSA and ECDC, 2020).

Dairy specialties made from unpasteurized milk pose a major concern considering that the manufacturing and ripening processes were demonstrated to be insufficient to obtain a complete inhibition of some STEC strains (Bellio et al., 2018; Ioanna et al., 2018). Here, we describe the characterization and comparative genomic analysis of seven STEC strains isolated from a semi-hard raw milk cheese, with the attempt to comprehend the virulence potential in a risk assessment perspective.

## Materials and Methods

### Sampling and Microbiological Analysis

Seventy-five semi-hard raw milk cheese samples were collected during a food company’s own quality check performed from April 2019 to January 2020 and analyzed following the standard procedure of UNI CEN ISO/TS 13136:2013. Briefly, 25 g of cheese was inoculated in 225 ml of buffered peptone water (BPW) and incubated at 37°C ± 1°C for 18/24 h. Then, 100 μl of the enrichment broth was processed for DNA extraction and real-time PCR using iQ Check STEC VirX Kit (Bio-Rad, Munich, Germany), following the manufacturer’s instructions. In order to verify the vitality of *E. coli* cells, the enrichment broth was streaked into Tryptone Bile X-glucuronide (TBX) plates incubated at 44°C ± 1°C for 18/24 h, and then, 50 colonies were isolated in nutrient agar (NA) and incubated at 37°C for 18/24 h. Using 10-colony pools, real-time PCR was performed in order to verify the presence of toxins. When one of this pool was positive, real-time PCR was performed on each single colony. A biochemical identification with API 20E test system (Biomerieux) was also performed on each colony with an *E. coli* profile following the manufacture’s procedure. Moreover, the enrichment broth was screened also by immunomagnetic separation of *E. coli* O157:H7 with Dynabeads *E. coli* anti-O157 kit (Invitrogen, Carlsbad, CA, United States). A loop from the bacteria–bead complex recovered after immunomagnetic separation (IMS) was inoculated onto MacConkey–Sorbitol agar base (CT-SMAC), spread plated, and incubated at 37°C for 24 h.

Stx-positive strains were subcultured in Violet Red Bile Agar (VRBA) plates at 37°C for 24 h, and a colony was picked up to be inoculated in Luria–Bertani (LB) broth. After overnight incubation at 37°C for 24 h, the culture was centrifuged, and the pellet was resuspended in 1 ml of LB broth supplemented with 20% of glycerol. Strains were stocked at −20°C for future use.

### DNA Extraction and Strain Characterization

For each sample, STEC colonies were streaked on VRBA plates (Oxoid) and incubated at 37°C for 24 h. Then, each colony was picked and inoculated in LB broth (Oxoid) incubated at 37°C for 24 h. Genomic DNA was isolated from 1 ml of an overnight culture by E.Z.N.A. ^®^ Bacterial DNA Kit (Omega Bio-tek), following the standard protocol. The DNA concentration was quantified using Qubit 2.0 Fluorometer (Thermo Fisher Scientific) and loaded in a 0.8% agarose gel in order to verify the DNA integrity.

### Whole Genome Sequencing and Assembly

Genomic DNA of seven strains (Table 1) was sequenced using Illumina Miseq platform (Illumina, Inc., San Diego, CA, United States) with 250 paired-end run after Nextera XT paired-end library preparation (Illumina). Raw sequence data quality was evaluated with FastQC software. Reads were assembled *de novo* with PATRIC web tool, discarding contigs with a length below the 300 bp (Davis et al., 2020), and contigs were annotated with Prokka with an e-value cutoff default (version 1.13.3).

**TABLE 1:** Genomic features of Shiga-toxin producing *Escherichia coli* (STEC) strains isolated in this study, divided by samples.

	Sample A			Sample B		Sample C	Sample D
Strain	UC4128	UC4129	UC4132	UC4130	UC4131	UC4133	UC4134
Size	5,062,922	4,819,408	5,062,789	5,063,194	5,651,041	4,820,057	4,765,942
GC%	50.7	50.6	50.7	50.7	50.2	50.6	50.6
N50	135,211	137,763	145,606	145,608	54,990	135,498	139,169
N. Contigs	148	88	146	138	381	95	97
N. CDS	4770	4543	4777	4773	5371	4544	4467
N. RNAs	93	97	93	94	103	97	97
ST #*E.coli*1	661	3519	661	661	661	3519	3519
Serotype	O174:H2	O116:H48	O174:H2	O174:H2	O174:H2	O116:H48	O116:H48
Clermont type	B1	A	B1	B1	B1	A	A

### Serotype, Multilocus Sequence Types, Virulence Profile, LAA PAI Identification, and Antimicrobial Resistance

The Center for Genomic Epidemiology (CGE) web tools^1^ were used to evaluate the *in silico* molecular characterization of the sequenced strains. Assemblies were analyzed with Serotype finder^2^ in order to define the O and H serotype, setting 85% of identity threshold and 60% of minimum length. The multilocus sequence type was determined using MLST Finder 2.0^3^ against the *E. coli* #1 set, including adenylate kinase (*adk*), fumarate hydratase (*fumC*), DNA gyrase (*gyrB*), isocitrate/isopropylmalate dehydrogenase (*icd*), malate dehydrogenase (*mdh*), *purA* (adenylosuccinate dehydrogenase), and *recA* (ATP/GTP binding motif) genes. Virulence Finder 2.0^4^ was used to search for virulence genes, setting an identity threshold of 90% with a minimum length protein of 60%. In the case of apparent incompleteness of a virulence gene, the integrity was confirmed with BLASTn analysis searching the complete sequence in two or more contigs. The following additional genes were searched using BLASTn, considering coverage and identity >90%: *saa* (AF399919.3; positions: 6290–7840 bp), *sab* (AY258503.2; positions: 118,905–123,200 bp), *lesP* (CP023541.1; positions: 4,063,646–4,067,740 bp), *tia* (JQ994271.1; positions: 4726–5472 bp), *hra1* (U07174.1), *ipaH* (M32063.1), *elt* (EU113248.1), *est* (M34916.1), *aggR* (Z18751.1), *aat* (AY351860), and *aaiC* (NC_008460.1; positions: 27,389–28,627 bp).

Geneious Prime v.2020.1 was used in order to map reads of the sequenced strains to LAA region reference sequence (AFDQ0100002; 385,984–472,336 bp) (Montero et al., 2017), generating a consensus used for annotating proteins with an 80% of similarity threshold. Genomes were also screened for antimicrobial resistance determinants with the Comprehensive Antibiotic Resistance Database (CARD; Alcock et al., 2020) and were analyzed to determine phylogroups with ClermonTyping^5^ (Beghain et al., 2018).

All the records obtained with CGE web tools and with CARD were verified with BLASTN and BLASTp databases [National Center for Biotechnology Information (NCBI)].

### Prophage and Plasmid Replicons

The number and the type of prophage sequences were predicted using Phage Search Tool Enhanced Release (PHASTER)^6^, identifying intact, questionable, and incomplete prophage sequences by scores of >90, 70–90, and <70, respectively (Arndt et al., 2016). In order to go deeply into the analysis, contigs of O174 strains were scaffolded using Multidraft-Based Scaffolder (MEDUSA)^7^ (Bosi et al., 2015). *E. coli* strain 89-3156 (NZ_CP027366.1) was used as complete reference genome.

The search of plasmid replicons was performed with PlasmidFinder v.2.1^8^ (Carattoli et al., 2014), and reads were also submitted to the PlasmidSPAdes service of Pathosystems Resource Integration Center (PATRIC) in order to separate plasmid contigs from genome contigs. The annotation of putative plasmid sequences was performed by Geneious Prime v.2020.1, setting a 80% similarity threshold.

### Phylogenetic Analysis

The draft genomes were analyzed with Roary (v3.11.2) (Page et al., 2015) to find out the similarity of isolated strains and the relationships between them. The Roary analysis identifies core genes as those shared by 95–99% of genomes, and accessory genes, which include shell genes found in 15–95% and cloud genes in <15% of genomes. After the cluster analysis, non-conserved accessory genes were analyzed, and similarities between groups were evaluated.

Other 21 genomes in FASTA format were downloaded from the NCBI database (data of download, 5 May 2020) and reannotated with Prokka. Roary was used to compare the cheese isolates with the reference genomes. Phylogenetic tree was visualized with Phandango^9^.

## Results

### STEC Isolation and Genomes Sequencing

When 75 semi-hard raw milk cheese samples were analyzed in the course of the company self-monitoring program, a total of 20 gave positive results in the *stx-*targeted PCR made on DNA extracted from the enrichment step, while all the samples were negative for *eae* gene. Further microbiological procedures and real-time PCR analysis were conducted in order to isolate and identify STEC colonies from the enrichment broth. Out of 20 enrichments, four samples (prevalence of 5.3%; IC 95%, 0.2–10.4%) were demonstrated to contain viable cells. In particular, we isolated a total of seven *stx*-positive colonies: three colonies from sample A, two colonies from sample B, one colony from sample C, and one colony from sample D (Table 1). The DNA of the seven strains was sequenced and assembled with a *de novo* approach. Table 1 describes the main characteristics of *de novo* genome assemblies. All samples had a coverage range of 88–169-fold and were assembled into 88–381 contigs. The GC content of all the analyzed strains was between 50.6 and 50.7% except for UC4131, which had a 50.2% value.

**FIGURE 1:**
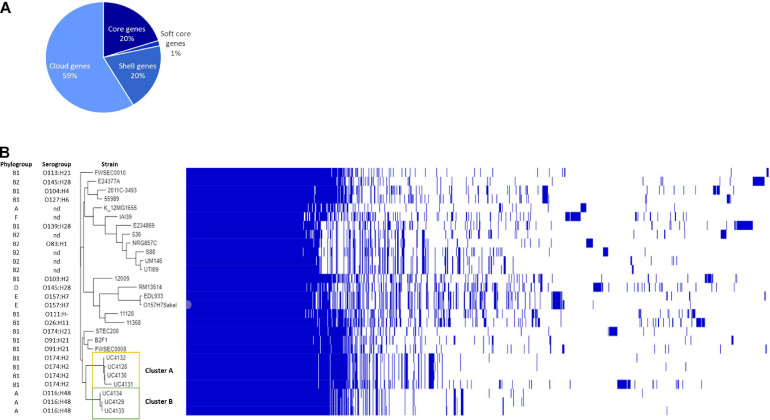
Genomic comparison between semi-hard raw milk cheese isolates and reference strains belonging to different pathotypes. **(A)** Pie chart represents the percentage of genes belonging to core (20%), soft core (1%) shell genes (20%), and cloud genes (59%); phylogenetic tree based on single-nucleotide polymorphism (SNP) detection in orthologous genes. **(B)** Each strain is characterized by the phylogroup and the serogroup. The figure shows the phylogenetic division of cheese isolates into two clusters (clusters A and B) and their correlation with other reference strains listed in Supplementary Table 1.

The first purpose of this study was to clarify if the *stx*-positive isolates from cheeses were different strains to avoid duplications in the study. The analysis with Roary recognized a pangenome of 5,903 genes, with a core genome of 4,121 genes, representing the 70% of all genes, and 1,782 accessory genes, of which 1,138 were classified by the software as shell genes (shared by 15–95%) and 664 as cloud genes (shared by 0–15%). In order to investigate the similarity between strains, accessory genes were considered. The phylogenetic analysis allowed to divide strains into two clusters (Figure 1B and Supplementary Figure 1). Cluster A was represented by UC4130, UC4128, UC4131, and UC4132 and was discriminated from cluster B, consisting of UC4129, UC4133, and UC4134, by a total of 1,288 genes, of which 628 were found in all the four strains of cluster A. Within this cluster, UC4130, UC4128, and UC4132 were differentiated by a limited number of unique genes (3–16). Differently, UC4131 exhibited 603 unique genes not found in any of the other analyzed strains. Among the 628 genes, 418 genes were hypothetical proteins, and the remaining genes belonged to membrane transport system, phage proteins (i.e., transposase), and proteins involved in plasmid replication and conjugation.

A total of 403 genes were typical of cluster B and absent in cluster A. The genes shared by the three strains were 327, and three to six unique genes were found. Out of 327 genes, 201 were recognized as hypothetical proteins, and the others consisted in carbohydrates metabolism, phage proteins, and membrane transport systems.

### *In silico* Serotyping, Multilocus Sequence Type, and Phylogroup

The genome sequence of all strains was used to determine the serotype, the sequence type, and the *in silico* Clermont typing, to better characterize the epidemiology of these *E. coli* strains, as shown in Table 1. The analysis with Serotype Finder tool identified two serogroups, O174:H2 (four strains) and O116:H48 (three strains). Sample A contained strains with both serogroups, while samples B–D included only one serogroup. Each serogroup corresponded to a sequence type, ST661 and ST3519, respectively. The Clermont phylogroup classification, consistent with ST and serogroup classification, divided strains in two groups, namely, phylogroup B1 for strains with ST 661 and phylogroup A for strains with ST 3519.

### *In silico* Determination of Virulence Profile, LAA PAI Region, and Antimicrobial Resistances

**FIGURE 2:**
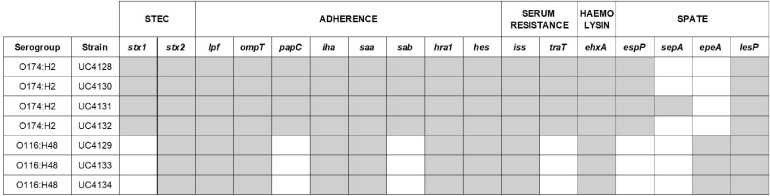
Virulence gene pattern analyzed in this study. The presence of virulence genes is identified with gray cells, the absence with white cells. Other virulence determinants involved in adherence (*tia* and *eibG*) and markers of *E. coli* pathotypes [EHEC/EPEC (*eae*), EIEC (*ipaH*), ETEC (*elt* and *est*), and EAEC (*aggR*, *aatA*, *aaiC*)] resulted absent in all the Shiga-toxin producing *Escherichia coli* (STEC) strains.

Whole genome sequences were interrogated to identify virulence genes using the database of Virulence Finder tool, and results are shown in Figure 2. While the applied real-time PCR was not able to distinguish the *stx* gene variants carried by isolated strains, the WGS analysis showed that all the strains carry the *stx2* gene of subtype a. In addition, O174:H2 serogroup strains harbor also the *stx*1 gene, as reported in Figure 2. The intimin coding gene *eae* was not detected in any of the analyzed strains. The virulence profile included genes coding for adherence factors, such as *lpfA* coding long polar fimbriae and *iha* coding IrgA homolog adhesin, outer membrane protein *ompT* and *PapC* gene involved in fimbrial adhesin system; the serum resistance factor genes *iss* and *traT*; and plasmid-encoded virulence genes, such as enterohaemolysin (*ehxA*). Strains that belonged to the same ST shared the same serine protease autotransporter system (SPATE) proteins. In particular, ST661 carried the *espP* gene, while *epeA* gene was typical of ST3519. The virulence pattern was examined in depth, searching for virulence determinants that can be found in LEE-negative strains of animal or human origin, which were recognized as makers of different pathotypes: *ipaH* as a marker of enteroinvasive *E. coli* (EIEC); *elt* and *est* as markers of enterotoxigenic *E.coli* (ETEC); and *aggR*, *aat*, and *aaiC* as markers of enteroaggregative *E. coli* (EAEC). None of these marker genes was found in the seven analyzed strains. Differently, all strains harbored genes coding for two other adherence factors, namely, *saa* gene and heat-resistant agglutinin 1 gene (*hra1*). The *sab* gene, encoding for STEC autotransporter, contributing to biofilm formation, was found only in ST661 strains. The overall pattern displayed by all strains was the same, considering that *espP*, *epeA*, *lesP*, and *sepA* are homolog members of the serine protease autotransporters of *Enterobacteriaceae* (SPATE) family.

**FIGURE 3:**
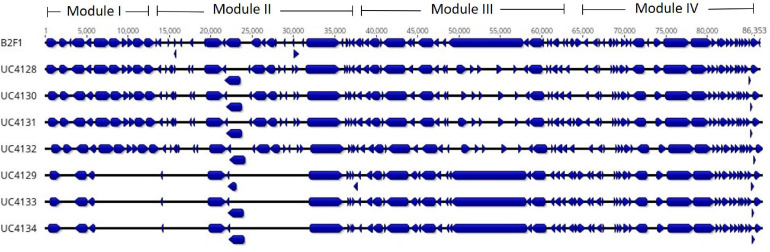
Identification of the locus of adhesion and autoaggregation pathogenicity islands (LAA PAI) in Shiga-toxin producing *Escherichia coli* (STEC) strains isolated from cheese and comparison with the LAA PAI of reference strain *E. coli* B2F1 (first line). Alignment shows the open reading frames (ORFs), identified by blue blocks. The figure shows the lack of ORFs in UC4129, UC4133, and UC4134 in modules I and II.

All strains were screened for the presence of LAA region, a pathogenicity island detected in LEE-negative STEC strains. The reference mapping and annotation against LAA region published by Montero et al. (2017) revealed the presence of this genomic island in each strain but with some differences. UC4128, UC4130, UC4131, and UC4132 strains showed an almost complete LAA region. As shown in Figure 3, they lacked a fragment of filamentous hemagglutinin gene (*tps* gene; WP_001081255.1), in the module III region, and transposase (WP_000088311.1 and WP_001367783.1). Contrariwise, UC4129, UC4133, and UC4134 exhibited almost incomplete modules I and II.

The resistome investigation with CARD did not lead to the detection of acquired genes coding for antimicrobial resistance determinants.

### Prophage and Plasmid Identification

**FIGURE 4:**
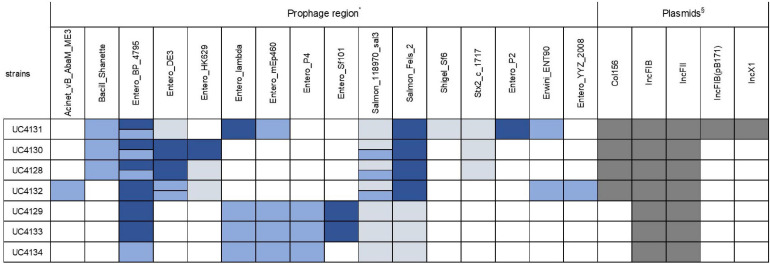
Type of prophage and plasmid replicons detected in all the sequenced strains. Intact prophage regions are evidenced in dark blue, while light blue and gray colors specify questionable and incomplete prophage regions, respectively. When the same prophage region is found in more than one copy, two bars are used to indicate the different homology with reference regions. Positivity for plasmid replicons are shown in dark gray. *Accession numbers of prophage sequences: Acinet_vB_AbaM_ME3 (NC_041884); Bacill_Shanette (NC_028983); Entero_BP_4795 (NC_004813); Entero_DE3 (NC_042057); Entero_HK629 (NC_019711); Entero_lambda (NC_001416); Entero_mEp235 (NC_019708); Entero_mEp460 (NC_019716); Entero_P4 (NC_001609); Entero_phiP27 (NC_003356); Entero_Sf101 (NC_027398); Pectob_ZF40 (NC_019522); Salmon_118970_sal3 (NC_031940); Salmon_Fels_2 (NC_010463); and Shigel_Sf6 (NC_005344); Stx2_c_1717 (NC_011357). §Accession number of plasmid replicons: Col156 (NC009781); IncFIB (AP001918); IncFII (AY458016); IncFIB (pB171) (AP001918); IncX1 (EU370913).

**FIGURE 5:**
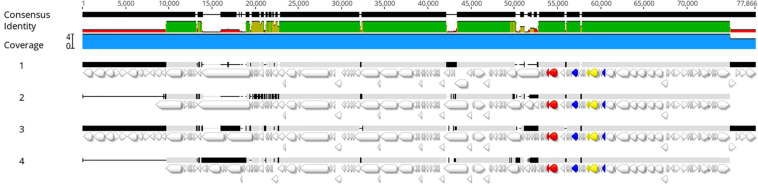
Alignment of prophage regions extracted from UC4128 (1), UC4130 (2), UC4131 (3), and UC4132 (4) genomes. The following genes are evidenced in different colors: *stx1* genes yellow, *stx2* red, and Q antiterminator sequences blue. The colored consensus identity line indicates the mean pairwise identity over all pairs in the column: green indicates 100% identity; greenish-brown indicates an identity between 30 and 100%, and red shows regions with identity below 30%. The upper black line shows sequences where a consensus is built on at least two sequences (thick bar) or regions where it is not possible to obtain a consensus sequence because of open gaps (thin bar).

All genomes were screened for the presence of prophage regions, a feature that represents the genome plasticity. Due to the presence of small contigs, *E. coli* strain 89-3156 (NZ_CP027366.1) complete genome was used to generate scaffolds for strains with ST661. A total of 59 prophage regions (Figure 4) with homology to 16 different phages were detected by PHASTER in the seven analyzed strains. Bioinformatic analysis identified 17 intact, 17 questionable, and 25 incomplete prophages. All the strains but UC4134 contained at least one complete prophage region. Most common phage regions were *Enterobacteria* phage BP-4795, *Salmonella* phage 118970_sal3, and *Salmonella* Enterica phage Fels-2, shared by all strains. For ST661 strains, contigs were joined in scaffolds, and the prophage structure was deeply explored. In all of them, *stx1* and *stx2* genes were found to be carried by complete prophages showing limited similarities (from 33.72 to 37.34%) with the *Enterobacteria* phage BP-4795. The analysis of the prophage sequences allowed identifying the attachment sites, namely, AttL and AttR, and typical prophage features, such as tail and head proteins, integrase, and lysis apparatus. Despite the same phage equipment, the length of the four prophages was quite different, ranging from 57 to 73 kb. As shown in Figure 5, the antitermination protein Q, required for the late transcription process, was found downstream *stx*1 and *stx2* genes, suggesting a hypothetical role in the regulation of *stx* gene expression.

The same analysis for ST3519 strains was not possible to be performed due to the lack of a complete reference genome, and, therefore, the search of prophagic sequences was done on contigs. In UC4129, the *stx2* gene was detected on a defective prophage, similar to the BP-4795 prophage (62,5% identity), lacking the attachment site, suggesting the impossibility of being an active phage. The same prophage was found as an incomplete sequence in UC4134, equipped with proteins belonging only to lysis and tail apparatus. A different situation concerned UC4133 because the *stx2* gene was carried by a very short contig that prevented an accurate prophage characterization.

Sequenced genomes were also screened to identify plasmid sequences and evaluate the possibility that these replicons harbored virulence genes. Two types of plasmid replicon sequences, belonging to IncFIB and IncFII groups, were detected in all the strains (Figure 4) located, respectively, in the same contig of *ehxA* gene and *iha* gene and therefore suggesting that this virulence factors were plasmid coded. In addition, the strain of ST661 harbored a Col156 replicon. The analysis of the UC4131, the ST661 strain with a larger genomes size (5.6 Mb), showed that this strain carries also IncFIB and IncX1 replicons and 47 contigs, corresponding to more than 495 kb that were recognized as plasmid sequences by PlasmidSpades software. This suggested that the larger genome size of this strain is attributable to the presence of large plasmids. Multialignment analysis of the ST3519 strains revealed that UC4129 and UC4134 carried the same 6.9 kb plasmid (identity of 100%) and that UC4133 shared a 95% of the same sequence. This plasmid showed high similarity with pColE2-P9 of *E. coli* BZB1011 E2C (KY348421.1), a plasmid that was demonstrated to confer the capability of dominating the other strains through the production of the bacteriocin Colicin E (Götz et al., 2018).

### Phylogenetic Comparison

The WGS of strains isolated from cheese were compared with reference genomes selected for their human origin and representing the most prevalent pathotypes and LEE-negative emerging clones (Supplementary Table 1). Pangenome was composed of a total of 14,718 genes, of which 20% represented the core genome. Among the accessory genes, 8,632 predicted open reading frames (ORFs) were “cloud genes,” namely, genes shared by 1–15% of analyzed strains (Figure 1A). Phylogenetic analysis evidenced that cheese isolates clustered with LEE-negative emerging STEC STEC200, FWSEC0008, and B2F1 strains and are separated by the other reference strains (Figure 1B).

## Discussion

Recently, concern about foodborne STEC infections has increased, and the analysis of “strong evidence” outbreaks pictured raw milk cheese as one of the major sources of STEC. In particular, six outbreaks were related to the consumption of milk and dairy products in 2012–2017 (EFSA and ECDC, 2020). The persistence of STEC strains from raw milk during the cheese-making process and along the aging period has been already demonstrated (Caro and García-Armesto, 2007; Rios et al., 2020).

Here, we described the isolation of STEC strains from a semi-hard raw milk cheese, with a prevalence of 5.3%. Our results were consistent with previous data available about other raw milk cheeses, reporting different prevalence of STEC and ranging from 3.7% in 2006 to 6.3% in 2007 in Switzerland (Stephan et al., 2008), 11.3% in Egypt (Elhadidy and Mohammed, 2012), 17.5% in Iran (Mohammadi et al., 2013), and 13% in France (Vernozy-Rozand et al., 2005).

Whole genome sequencing is recognized as a rapid and useful tool to provide a deep genomic characterization from a foodborne pathogen surveillance point of view (Ronholm et al., 2016). In this study, WGS analysis was performed in order to investigate the genomic features and the virulence profile of the seven isolated strains. Among the seven strains, two serotypes, O174:H2 an O116:H48, were detected. These serogroups appear to be emerging in the last years in the STEC landscape. O174 is one of the serogroups that was included in the top 20 most frequent serogroups identified as causing human infection and was found in the last 7 years mainly in bovine and goat meat with a frequency below of 1% (EFSA and ECDC, 2019). Only a survey on 400 raw milk cheese samples identified a single strain with O174:H2 serotype (Madic et al., 2011). The other serotype O116 was reported to be isolated from meat with a very low prevalence (<0.1%) (EFSA and ECDC, 2019), but no studies have linked it with dairy products. Noteworthy, the first sample, found to be *stx*-positive in April 2019, contained both serotypes, which were detected in samples analyzed in the following months. The detection of the same serotypes in different lots of this type of cheese may be due to the capability of these strains to be tolerant to the cheese-making and to the ripening process. It was observed by other authors that the capacity of some STEC strains to survive to the cheese manufacturing process (Bellio et al., 2018; Ioanna et al., 2018) can be a strain-dependent feature; this behavior should be confirmed by an artificial contamination test to verify the ability of these strains to persist in the final product. Interestingly, our study divided the seven analyzed strains into two groups, defined by the same serogroup, sequence type, and Clermont type. This evidence was confirmed by the phylogenetic analysis that divided them into two clusters. The investigation of accessory genes among the seven strains highlighted that all the cloud genes consisted of unique genes (*n* = 644), in which 603 belonged to UC4131 and the remnants to the other six strains. Since the differences among strains were due to a very low number of strain-specific genes, we hypothesized that they were very similar, sharing most of the genes, especially among the same cluster. The high number of unique genes of UC4131 and the estimation of a larger genome size makes this strain different from the others. This observation, together with the detection of a higher number of plasmid replicons and the 0.5 M-b sequences of plasmid origin, may induce to suppose that UC4131 harbors extrachromosomal elements.

To better characterize the virulence potential of the cheese isolates, the genomes of all the seven strains were screened for genetic determinants associated with pathogenicity. *In silico* genome analysis of cheese isolates confirmed that all these strains are STEC, with the difference that the three strains belonging to the O116 serotype carried the *stx2* gene while the four O174 serotypes carried both *stx1* and *stx2* genes. The presence of *stx2* gene is relevant because it is demonstrated to be associated with the development of human disease (Farrokh et al., 2013). The absence of the intimin coding *eae* gene in all the strains led to their exclusion to the enterohemorrhagic *E. coli* (EHEC) group. Up to now, a specific virulence pattern unambiguously correlated with the development of illness was not defined, but some virulence determinants were recognized as more frequently associated with *E. coli* pathotypes (Robins-Browne et al., 2016). Among seven strains, no typical pathogenicity markers were detected, indicating that these strains do not belong to the most common pathotypes. An exception is represented by the enterohemolysin coding gene *ehxA*, frequently detected on plasmids in EHEC strains and frequently reported to be associated with *stx* genes (Beutin et al., 1989; Peng et al., 2019) and with the possibility to develop severe diseases (Franz et al., 2015). Moreover, the investigation of other virulence determinants showed the presence of adhesins and genes belonging to the serine protease autotransporters of Enterobacteriaceae (SPATE) family in all strains. Their important role in the adherence to intestinal epithelial cells and in the biofilm formation together with the involvement in the colonization process was already demonstrated (Orth et al., 2010; Monteiro et al., 2016; Krause et al., 2018). It is not known, however, if the biofilm formation ability exerts a role in the persistence in the environments, such as the cheese-making facilities and production plants.

Some reports evidenced that LEE-negative strains are emerging pathogens. Consistent with the study of Montero et al. (2017, 2019), we observed LAA PAI in our LEE-negative strains. In particular, an almost complete LAA PAI was found in four strains, while the other strains lacked two modules. The presence of complete LAA PAI represented an important factor, since LAA PAI positive strains were isolated from severe disease cases (Montero et al., 2017, 2019). A recent study demonstrated the participation of LAA-PAI to the intestinal colonization in mouse model, contributing to confirm the relevance of this genetic island in a pathogenicity perspective (Montero et al., 2019). The genomic correlation of our strains with LEE-negative strains was proved by phylogenetic comparison, which clustered our strains with other emerging LEE-negative strains.

Genomic characterization led to identification of the presence of plasticity elements in all strains, such as plasmid replicons and phage regions. Our study confirmed the evidence that some virulence genes, like *iha* and *ehxA*, were carried by plasmids, promoting their dissemination to other strains in the same environment. It was not surprising to find prophage regions associated with *stx* genes, confirming the link between prophage Entero BP 4795 and Shiga toxins (Creuzburg et al., 2005; Zhang et al., 2020) in the case of strains belonging to the ST661. The presence of antiterminator Q downstream to *stx* genes, already studied for their ability to promote the transcription of Shiga toxins, was observed in BP-4795-like prophage sequence of UC4128, UC4130, UC4131, and UC4132 genomes, opening to the possibility of *stx* expression in certain conditions. Further studies will be performed to explore this hypothesis. Consistent with previous research, a good variability among the sequence of prophagic regions was observed, providing the evidence that isolates are different strains (Gamage et al., 2004).

This paper confirmed the efficacy of the WGS analysis as a tool for studying the virulence potential of STEC strains isolated from food. This approach has allowed to characterize strains from two persistent serotypes, O174 and O116, that have been isolated over a 8-month period from ripened cheese. The data acquired indicated the presence in cheese of *E. coli* strains harboring the *stx* phages and non-LEE pathogenicity island. Further studies are still necessary to increase the knowledge on the persistence of STEC in livestock and milk-derived products and to design mitigation strategies at farm and cheese manufacturing level to assure the safety of the final product.

## Data Availability Statement

The datasets presented in this study can be found in online repositories. The names of the repository/repositories and accession number(s) can be found below: https://www.ncbi.nlm.nih.gov/ PRJNA666781.

## Author Contributions

MB carried out microbiological experiments. CC performed the genome analysis. PC, CC, and DB designed the experiments and contributed to the interpretation of results. CC wrote the manuscript in consultation with PC and DB. All authors discussed the results and critically revised and approved the final manuscript.

## Conflict of Interest

The authors declare that the research was conducted in the absence of any commercial or financial relationships that could be construed as a potential conflict of interest.

## Footnotes

1 http://www.genomicepidemiology.org/

2 https://cge.cbs.dtu.dk/services/SerotypeFinder/

3 https://cge.cbs.dtu.dk/services/MLST/

4 https://cge.cbs.dtu.dk/services/VirulenceFinder/

5 http://clermontyping.iame-research.center/

6 https://phaster.ca/

7 http://combo.dbe.unifi.it/medusa

8 https://cge.cbs.dtu.dk/services/PlasmidFinder/

9 https://jameshadfield.github.io/phandango/#/
