# Supplementary material for: Genomic Investigation of Virulence Potential in Shiga Toxin Escherichia coli (STEC) Strains From a Semi-Hard Raw Milk Cheese
Source: Front Microbiol. 2021 Feb 1;11:629189. doi: 10.3389/fmicb.2020.629189 (PMC7882498; doi:10.3389/fmicb.2020.629189)

**Supplementary information**

**Supplementary Table 1**. Main characteristics of reference genomes used for the genomic comparison with STEC isolates from raw-milk cheese (* LEE-negative emerging strains).

| **strain** | **serotype** | **pathotype** | **Assembly level** | **Genome size(Mb)** | **isolation source** | **accession number** |
| --- | --- | --- | --- | --- | --- | --- |
| **2011C-3493** | O104:H4 | STEC/EAEC | complete | 5.27 | HUS | NC_018658.1 |
| **11368** | O26:H11 | STEC | complete | 5.70 | diarrhea | NC_013361 |
| **B2F1*** | O91:H21 | STEC | contig | 5.00 | HUS | GCA_000234255.3 |
| **12009** | O103:H2 | STEC | complete | 5.52 | sporadic cases of diarrhea/bloody stool | NC_013353 |
| **11128** | O111:H- | STEC | complete | 5.77 | sporadic cases of diarrhea/bloody stool | NC_013364 |
| **Sakai** | O157:H7 | STEC | complete | 5.59 | HUS | NC_002695 |
| **EDL933** | O157:H7 | STEC | complete | 5.55 | HUS | CP008957.1 |
| **55989** | O127:H6 | EAEC | complete | 5.15 | nd | NC_011748 |
| **E2348/69** | O139:H28 | EPEC | complete | 5.07 | infantile diarrhea outbreak | NC_011601 |
| **E24377A** | O145:H28 | ETEC | complete | 4.98 | diarrhea | NC_009801 |
| **RM13514** | O145:H28 | STEC | complete | 5.59 | lettuce-associated outbreak | CP006027.1 |
| **UTI89** | nd | ExPEC | complete | 5.07 | patient with an acute bladder infection | NC_007946 |
| **536** | nd | ExPEC | complete | 4.94 | uropathogenic *Escherichia coli* | NC_008253 |
| **S88** | nd | ExPEC | complete | 5.03 | meningitis | NC_011742 |
| **K12** | nd | commensal | complete | 4.64 | nd | NC_000913 |
| **NRG 857C** | O83:H1 | AIEC | complete | 4.75 | Crohn's disease patient | NC_017634 |
| **UM146** | nd | AIEC | complete | 4.99 | Crohn's disease patient | NC_017632 |
| **IAI39** | nd | UPEC | complete | 5.13 | Urine of patient with pyelonephritis | NC_011750 |
| **FWSEC0008*** | O91:H21 | EHEC | complete | 4.97 | clinical | GCA_005037775.2 |
| **FWSEC0010*** | O113:H21 | STEC | complete | 4.96 | clinical | NZ_CP031898.1 |
| **STEC 200*** | O174:H21 | STEC | scaffold | 5055076 | diarrhea | GCA_001607435.1 |

**Supplementary Figure 1.** Phylogenetic tree inferred from core genes alignment of cheese isolates, characterized by Sequence Types and Serogroups. The seven strains are divided into two clusters, Cluster A and Cluster B.


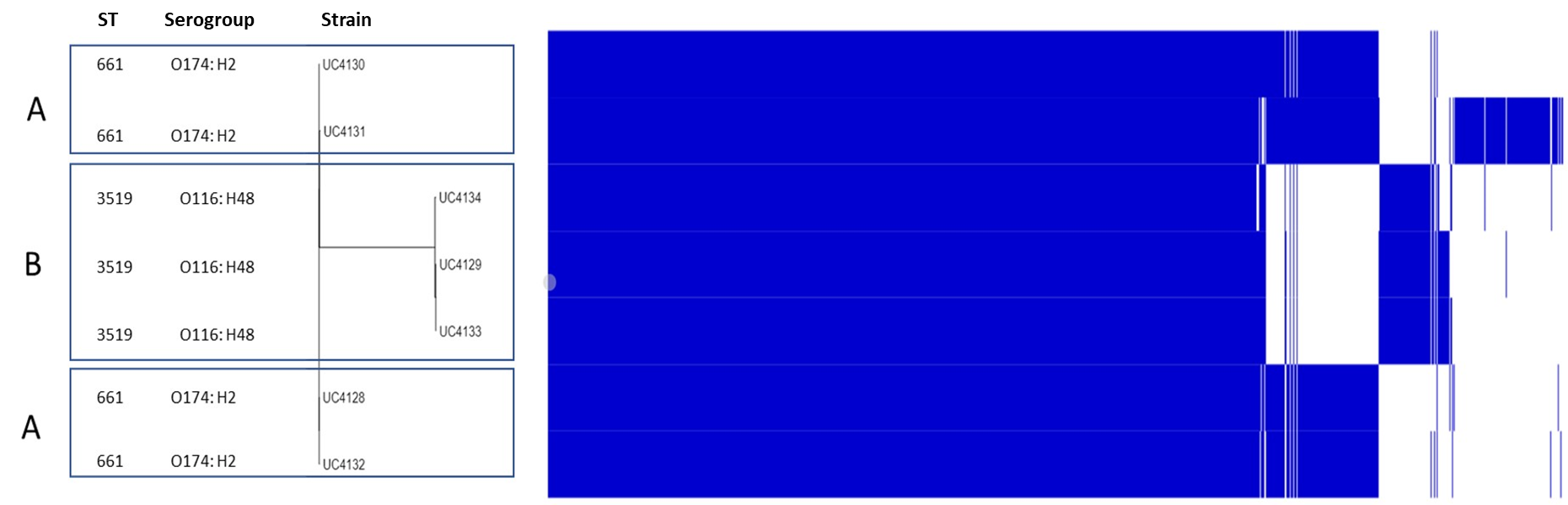

Supplement: Supplementary file 1 [file Data_Sheet_1.doc]
